# Rediscovering parainfectious encephalopathy in the post-COVID-19 era

**DOI:** 10.3389/fimmu.2025.1634383

**Published:** 2025-09-25

**Authors:** Lin Bai, Chang Geng, Hongzhi Guan

**Affiliations:** Department of Neurology, Peking Union Medical College Hospital, Chinese Academy of Medical Sciences and Peking Union Medical College, Beijing, China

**Keywords:** parainfectious encephalopathy, COVID-19, cytokines, autoinflammatory, innate immunity

## Abstract

The COVID-19 pandemic has unveiled the pivotal role of systemic inflammatory responses in neurological complications, particularly parainfectious encephalopathy. Accumulating evidence has established innate immune overreaction—distinct from direct viral neuro-invasion or autoantibody-mediated reaction—as the fundamental mechanism. The clinical manifestations of parainfectious encephalopathy are highly diverse, spanning from mild cases, such as mild encephalopathy with or without a reversible splenial lesion (MERS or ME), to catastrophic syndromes like acute necrotizing encephalopathy (ANE) and febrile infection-related epilepsy syndrome with or without a claustrum lesion (FIRES-C or FIRES). In this article, we summarize the phenotypes, diagnosis, and treatment strategies for parainfectious encephalopathy to enhance clinical recognition and understanding of this re-emerging disorder.

## 1 Introduction

During the COVID-19 pandemic in the past few years, parainfectious encephalopathy has become a key focus in neuroinfectious and neuroimmunological research. For neurologists, the initial clinical issue is whether SARS-CoV-2 causes viral encephalitis or possesses neuroinvasive properties. Accumulating evidence from systematic and in-depth research has progressively clarified our understanding of the underlying mechanisms.

## 2 COVID-19-associated encephalopathy: a parainfectious encephalopathy

COVID-19-associated Encephalopathy is driven primarily by systemic inflammatory cascades rather than direct viral invasion of the central nervous system (CNS). Zamani et al. (1) analyzed 182 COVID-19 cases with CNS involvement, finding no clinical or ancillary evidence of direct neural infection. Neuropathological studies support this conclusion: autopsies from the United States and Germany detected SARS-CoV-2 only in vascular endothelial cells in the brain, with no evidence of viral invasion into neurons or glial cells. Other pathological findings included glial activation, microglial nodules, and mild inflammatory infiltration (predominantly by monocytes and T lymphocytes) (2, 3). A Chinese cohort study demonstrated blood-brain barrier disruption (reflected by reduced GFAP immunoreactivity) without neuronal viral RNA detection (4). These changes resemble influenza virus-associated encephalopathy, where GFAP staining shows clasmatodendrosis—fragmentation and loss of astrocytic processes. Additionally, cerebral edema, necrosis, and hemorrhage were prominent, yet inflammatory cell infiltration was minimal (5).

**Figure 1:**
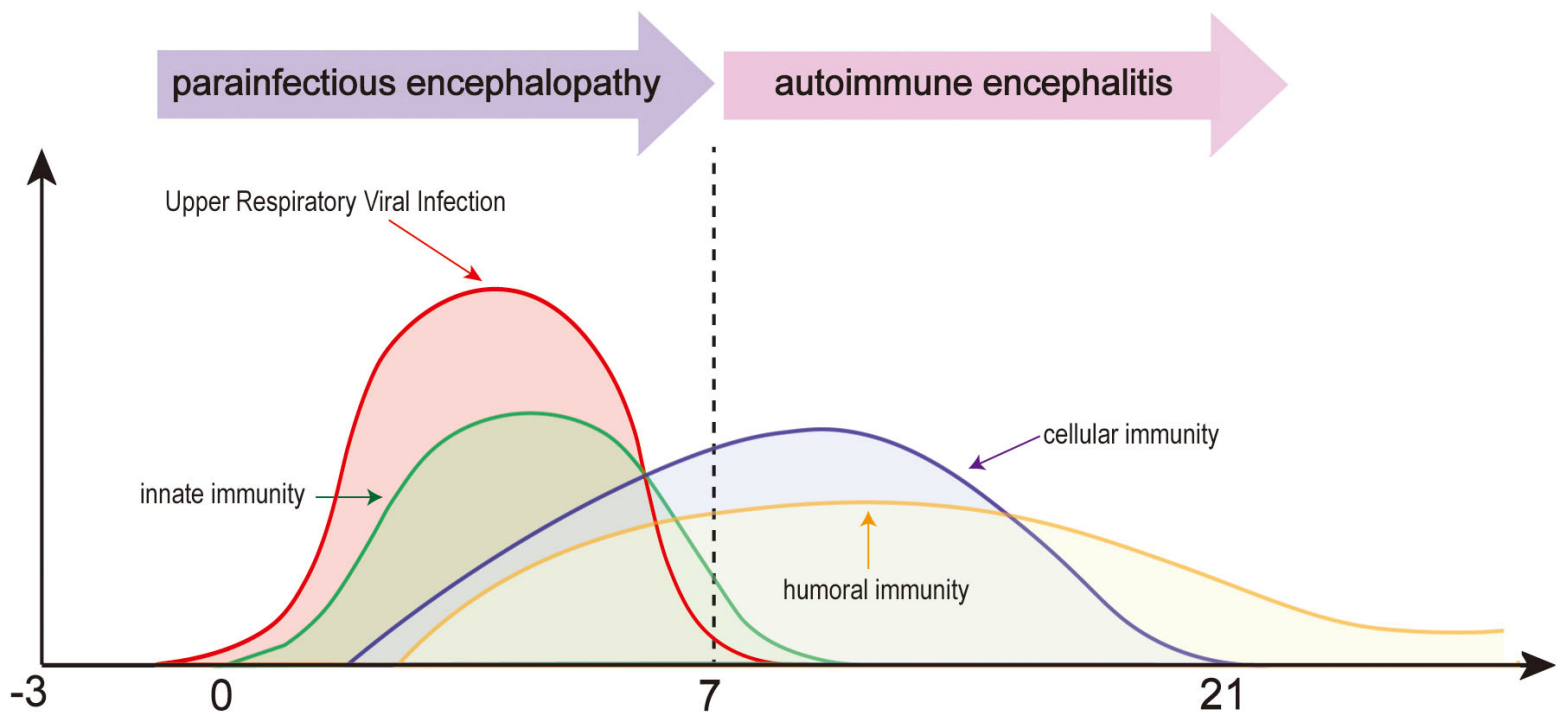
Temporal dynamics of parainfectious and autoimmune encephalitis following upper respiratory viral infection.

Encephalopathy caused by respiratory viruses such as SARS-CoV-2 is distinct from viral encephalitis caused by neurotropic viruses or autoimmune encephalitis mediated by specific antibodies. Instead, it is best categorized as a distinct third type—parainfectious encephalopathy. Respiratory viral infections can trigger a rapid innate immune response, leading to cytokine storms and systemic inflammatory syndrome. These conditions reflect CNS involvement in systemic inflammatory responses (6) (**Figure 1**). In the 1990s, Japan reported a series of influenza A–associated parainfectious encephalopathies, including acute necrotizing encephalopathy (ANE), which showed similarities to COVID - 19-related parainfectious encephalopathy in clinical presentation, neuroimaging features, and neuropathology (7). In recent years, we have encountered a range of parainfectious encephalopathy cases in clinical practice. Based on accumulating experience and literature review, we propose a preliminary classification of parainfectious encephalopathy subtypes.

## 3 Clinical phenotypes of parainfectious encephalopathy

### 3.1 Mild encephalopathy

During the acute phase of respiratory viral infections, some patients may experience transient confusion or delirium accompanied by fever, with normal neuroimaging, electroencephalography (EEG), and cerebrospinal fluid (CSF) analysis. After excluding potential causes such as hypoxia, ischemia, and metabolic disturbances, this self-limiting mild encephalopathy can be classified as reversible mild encephalopathy. Some researchers have proposed the term “benign variant of encephalopathy”, distinguishing it from other severe subtypes of parainfectious encephalopathy (8).

### 3.2 Mild encephalopathy with a reversible splenial lesion

**Figure 2:**
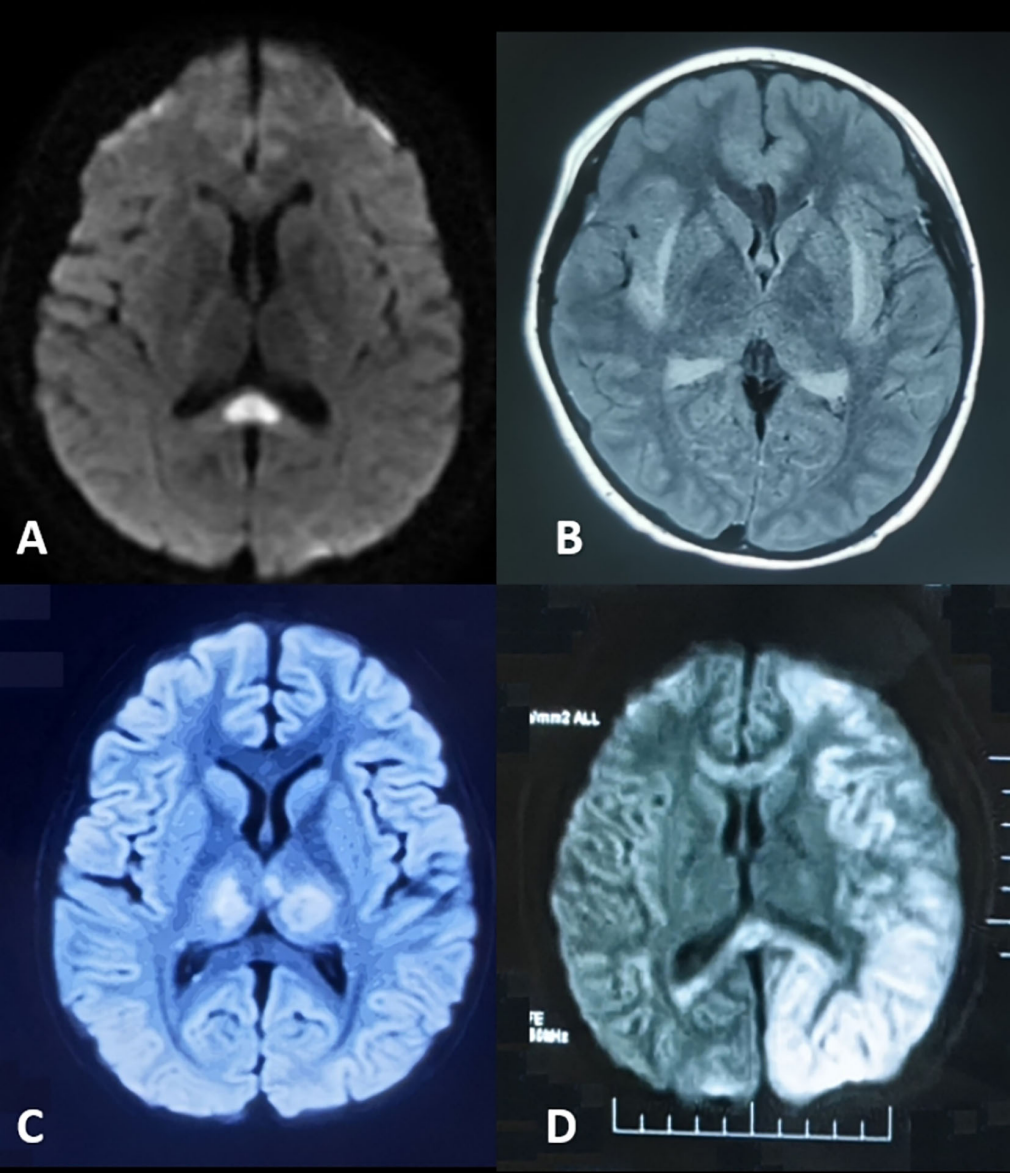
Neuroimaging of parainfectious encephalopathies. **(A)** Mild Encephalopathy with Reversible Splenial Lesion: splenium of the corpus callosum hyperintensity on diffusion-weighted imaging (DWI). **(B)** Febrile Infection-Related Epilepsy Syndrome with Claustrum Lesion: bilateral claustrum hyperintensity on T2-weighted and fluid-attenuated inversion recovery (FLAIR) imaging. **(C)** Acute Necrotizing Encephalopathy: bilateral thalamic hyperintensity on T2/FLAIR imaging. **(D)** Hemiconvulsion-Hemiplegia Syndrome: left cerebral cortex and subcortical hyperintensity on DWI with corpus callosum involvement and associated edema.

The neuroimaging hallmark of this condition is reversible corpus callosum splenium lesions, manifesting as diffusion-weighted imaging (DWI) hyperintensity with restricted diffusion (**Figure 2A**). First described by Tada et al. (9) in 2004, MERS was initially identified in a case series predominantly involving Japanese children and adolescents. The etiology of MERS is multifactorial. While early cases were primarily associated with influenza and rotavirus, recent evidence implicates SARS-CoV-2 as an additional trigger (10).

### 3.3 Febrile infection-related epilepsy syndrome

Previously termed *Febrile Illness-Related Epilepsy Syndrome*. FIRES predominantly affects children, manifesting as seizure onset within 24 hours to several days after a prodromal infection (typically respiratory viral infections), with rapid progression to status epilepticus. The acute-phase mortality rate reaches up to 30%, with survivors frequently developing long-term sequelae such as drug-resistant epilepsy and cognitive impairment (11). The acronym “FIRES” aptly captures the disease’s devastating and rapid course. However, the absence of specific diagnostic biomarkers poses significant challenges to timely diagnosis and management.

### 3.4 Febrile infection-related epilepsy syndrome with claustrum lesion

FIRES-C is a distinct subtype of FIRES, marked by claustrum abnormalities on neuroimaging (**Figure 2B**), which may serve as a diagnostic biomarker. In a cohort reported by Lin Bai et al. (12), 65% of patients were female, with a median age of 20.5 years. The median interval from fever onset to seizures was 5 days. Most presented with generalized tonic-clonic seizures; 45% experienced transient psychiatric or behavioral symptoms, and 50% required ICU admission. MRI typically revealed bilateral claustral lesions, sometimes accompanied by hippocampal signal abnormalities, usually detected around 12.5 days after symptom onset. CSF showed normal or mildly elevated white cell counts. Despite intensive immunotherapy, prognosis was poor, with 58% developing refractory epilepsy at one-year follow-up.

### 3.5 Acute necrotizing encephalopathy

ANE is characterized by rapid-onset encephalopathy, seizures, and focal neurological deficits, often following a febrile illness. Geng Chang et al. reported that in the Chinese population, symptoms typically develop 1~6 days (median 2 days) after respiratory viral infections, presenting as rapidly progressive consciousness disturbances and seizures (13). The hallmark imaging feature is bilateral thalamic lesions (**Figure 2C**), often accompanied by involvement of supratentorial gray and white matt, brainstem, and cerebellum. Elevated CSF IL - 6 levels and RANBP2 gene abnormalities are key diagnostic markers. ANE is associated with high mortality and disability rates. However, early aggressive immunotherapy may improve outcomes. As a prototypical parainfectious encephalopathy, ANE has been extensively studied in China, especially during COVID-19, advancing our understanding of its mechanisms and management (14–16).

### 3.6 Acute encephalopathy with biphasic seizures and late reduced diffusion

AESD was first described by Takanashi et al. in 2008 (17). This East Asia-prevalent disorder predominantly affects infants and young children. Zhang Meijiao et al. reported 21 pediatric cases presenting biphasic progression: initial seizures within 24 hours post-febrile onset, a quiescent period of 3~5 days, followed by recurrent seizures or altered consciousness from days 3 to 7. Characteristic DWI findings (days 3~14) demonstrate subcortical/cortical hyperintensity (“bright tree appearance”), while follow-up MRI reveals frontal/frontoparietal atrophy or residual lesions. Atypical monophasic cases have also been documented (18).

### 3.7 Hemiconvulsion-hemiplegia syndrome

HHS typically occurs in infants and young children. It is characterized by prolonged unilateral clonic seizures during febrile illnesses, followed by persistent hemiplegia. MRI reveals diffuse T2 hyperintensity and restricted diffusion in the affected hemisphere, often with severe edema (**Figure 2D**). Within one month, the affected hemisphere may show atrophy and hippocampal sclerosis. Approximately 80% of patients develop focal epilepsy.

### 3.8 Encephalopathy with malignant edema

It presents with acute psychiatric symptoms, refractory seizures, impaired consciousness, and coma. CT and MRI reveal extensive white matter lesions and cerebral edema. Cases have been reported in both influenza A and COVID-19 infections, with a very poor prognosis (8, 19, 20).

### 3.9 Meningism (Pseudomeningitis)

Meningism manifests meningeal irritation during acute febrile illness. In 1938, Merritt et al. reported 70 cases (21), mostly in individuals under 20, characterized by acute headache, neck stiffness, and a positive Kernig’s sign. CSF findings typically showed normal leukocyte counts with elevated intracranial pressure. The condition was self-limiting. Fishman later described meningismus as a diagnosis of exclusion, requiring differentiation from viral meningitis (22).

### 3.10 Complex type of parainfectious encephalopathy

Multiple clinical phenotypes may coexist in a single patient. In some cases, patients present with diffuse lesions resembling acute disseminated encephalomyelitis (ADEM); however, pathological findings often reveal minimal perivascular lymphocytic infiltration and absence of classic perivenous inflammation, distinguishing them from typical ADEM or other demyelinating diseases of the CNS (23).

## 4 Diagnosis and differential diagnosis of parainfectious encephalopathy

### 4.1 Diagnostic key points for parainfectious encephalopathy

- Neurological or psychiatric symptoms (e.g., encephalopathy, seizures, or altered consciousness) emerging in the acute phase (1 day to several days) of systemic infections (mainly acute respiratory viral infections).
- CSF WBC count normal or mildly elevated, with elevated cytokines (e.g.,IL-6) in CSF and/or serum, and negative anti-neuronal antibodies.
- Distinct imaging features for subtypes like ANE, FIRES-C, MERS, HHS, and AESD.
- Reasonable exclusion of other causes.

### 4.2 Differential diagnosis of parainfectious encephalopathy

ANE, FIRES-C, MERS, AESD, and HHS exhibit distinct clinical and neuroimaging phenotypes (**Table 1**), making them recognizable and diagnosable. In contrast, other subtypes of parainfectious encephalopathy lack specific features and pose greater diagnostic challenges, necessitating thorough differential diagnosis (**Table 2**).

**Table 1:** Clinical phenotypes and imaging features of parainfectious encephalopathy.

Clinical Phenotypes	Imaging Features
Mild Encephalopathy	no specific
MERS	transient DWI hyperintensity in SCC
FIRES	no specific
FIRES-C	bilateral claustrum lesions
ANE	bilateral thalamic lesions with/without supratentorial white and gray matter, brainstem or cerebellar involvement
AESD	“Bright Tree Appearance” on DWI
HHS	diffuse hemispheric T2 hyperintensity with restricted diffusion and edema
Encephalopathy with Malignant Edema	extensive white matter lesions and cerebral edema
Meningism	no specific
Complex type	heterogeneous and complex manifestations

MERS, mild encephalopathy with a reversible splenial lesion; FIRES, febrile infection related epilepsy syndrome; FIRES-C, febrile infection related epilepsy syndrome with claustrum lesion; ANE, acute necrotizing encephalopathy; AESD, acute encephalopathy with biphasic seizures and late reduced diffusion; HHS, hemiconvulsion-hemiplegia syndrome; CAME, COVID - 19-associated monocytic encephalitis; SCC, splenium of the corpus callosum; DWI: diffusion-weighted imaging.

**Table 2:** Differential diagnoses for parainfectious encephalopathy.

a. hypoxic-ischemic encephalopathy
b. viral and autoimmune encephalitis
c. ADEM and other inflammatory demyelinating diseases of the central nervous system
d. metabolic encephalopathy
e. osmotic demyelination syndromes
f. drug-induced encephalopathy

ADEM, acute disseminated encephalomyelitis.

## 5 Immunotherapy for parainfectious encephalopathy

Intensive immunotherapy is crucial in severe parainfectious encephalopathies, particularly ANE and FIRES-C. First-line treatment typically includes high-dose glucocorticoid pulse therapy combined with intravenous immunoglobulin (IVIG). Our data confirm this regimen significantly improves neurological recovery in COVID - 19-associated ANE (14). Tocilizumab, an IL - 6 receptor antagonist, serves as a cornerstone therapeutic agent for COVID - 19-associated inflammatory syndromes. Given that parainfectious encephalopathy manifests as a component of systemic inflammatory response, this biologic agent demonstrates emerging therapeutic potential in severe encephalopathy subtypes, with expanding clinical applications (24). Our systematic review of tocilizumab-treated ANE cases revealed significantly improved neurological outcomes when administered within 24 hours of hospital admission (PROSPERO, ID CRD420251017817).

Parainfectious encephalopathy stems from early innate immune dysregulation and typically does not require long-term immunotherapy. Long-term therapies targeting adaptive immunity (e.g., rituximab, mycophenolate mofetil) have shown limited benefit in FIRES-C (12). Prolonged tocilizumab treatment may be effective for seizure control in some cases of FIRES-C; however, this requires further validation through cohort studies. The optimal dosing, frequency, and duration of tocilizumab remain under investigation. Based on our clinical experience, we have shortened the dosing interval from every four weeks to every two weeks to achieve a more rapid therapeutic effect.

## 6 Parainfectious encephalopathy and long neuro-COVID-19

The sequelae of parainfectious encephalopathy may be mechanistically associated with long neuro-COVID-19 syndrome. The long-term neurological complications of SARS-CoV-2 infection impose a substantial burden on affected patients. Proposed mechanisms include immune dysregulation, persistent inflammation, vascular dysfunction, and neurotransmitter imbalance (25). Notably, even patients with mild initial neurological symptoms demonstrate brain changes on structural MRI, particularly in white matter regions, which correlate with specific cognitive deficits (26). Recent studies by Schwabenland et al. have further demonstrated persistent neuroinflammation with distinct microglia-T-cell interactions in COVID - 19 brains (27).

Although parainfectious encephalopathy typically presents as acute or transient neurological damage, its sequelae may share similarities with many symptoms of long COVID - 19, including anxiety, memory loss, and cognitive impairment (“brain fog”). In clinical practice, we have observed that some FIRES-C patients with disease courses exceeding 6 months continue to respond well to tocilizumab, suggesting that sustained inflammatory activation may persist beyond the acute phase of parainfectious encephalopathy.

In summary, parainfectious encephalopathy is a group of autoinflammatory encephalopathy closely related to innate immune response. Its pathogenic mechanism, clinical phenotype, and immunotherapy are different from those of autoimmune encephalitis mediated by adaptive immune response. Parainfectious encephalopathy comprises a spectrum of acute encephalopathy syndromes triggered by systemic infections, characterized by heterogeneous clinical manifestations. For severe parainfectious encephalopathy cases, early diagnosis followed by aggressive immunotherapy is critical to improve outcomes. As research advances, accumulating clinical evidence will pave the way for a deeper, evidence-based consensus on its diagnosis and management.
